# Supplementary material for: The Effect of A Whey-Protein and Galacto-Oligosaccharides Based Product on Parameters of Sleep Quality, Stress, and Gut Microbiota in Apparently Healthy Adults with Moderate Sleep Disturbances: A Randomized Controlled Cross-Over Study
Source: Nutrients. 2021 Jun 27;13(7):2204. doi: 10.3390/nu13072204 (PMC8308271; doi:10.3390/nu13072204)
Supplement: Supplementary file 1 [file nutrients-13-02204-s001.zip › nutrients-1224298-SI.pdf]

Supplementary Material

The effects of a whey-protein and galacto-oligosaccharides based product on parameters of sleep quality, stress, and gut microbiota in apparently healthy adults with moderate sleep disturbances: a randomized controlled cross-over study

Anne Schaafsma[1*], Leonard Mallee[1], Maartje van den Belt[2], Ester G. M. Floris-Vollenbroek[2], Guus Kortman[2], Jouke Veldman[1], Daan van den Ende[3], Alwine Kardinaal[2].

***Table S1**: Changes in PSQI and absolute PSQI values per study group per period and for ITT, PP and modPP.*

Data are presented as mean ± SD. [1] Based on the independent samples T-test. [2] One-way repeated measures ANOVA with Bonferroni post-hoc analysis. [3]Mann Whitney U-test. [4] Related-samples Friedman's Two-way Analysis of Variance by Ranks. DP: dairy-based test product; Placebo: skimmed milk powder; d: day; PSQI: Pittsburgh Sleep Quality Index

	**Period 1**		*p-value[1]*	**Period 2**		*p-value*
	**DP**	**Placebo**		**DP**	**Placebo**	
**ITT**						
Change d0-d7	-0.80 ± 3.19	-1.00 ± 2.91	*0.787*	-0.65 ± 3.37	-0.26 ± 2.32	*0.576*
Change d0-d14	-0.89 ± 3.14	-1.44 ± 3.59	*0.496*	-0.97 ± 2.53	0.60 ± 2.80	***0.017***
Change d0-d21	-0.37 ±3.31	-1.06 ± 3.53	*0.407*	-0.56 ± 2.81	0.31 ±2.58	*0.183*
Time effect[2]	*0.492*	*0.673*		*0.674*	*0.219*	
Post-hoc analysis	*>0.709*	*>0.462*		*1.000*	*>0.442*	
PSQI d0	9.94 ± 2.55	10.62 ± 2.93	*0.278*	9.71 ± 3.25	9.23 ± 3.03	*0.530*
PSQI d7	9.14 ± 3.13	9.62 ± 3.24	*0.476*	9.33 ± 2.69	8.97 ± 3.63	*0.644*
PSQI d14	9.06 ± 2.75	9.18 ± 3.26	*0.870*	8.74 ± 2.49	9.83 ± 2.98	*0.103*
PSQI d21	9.57 ± 2.91	9.56 ± 2.96	*0.986*	9.15 ± 3.27	9.54 ± 3.12	*0.608*
Time effect[2]	*0.270*	*0.069*		*0.284*	*0.285*	
Post-hoc analysis	*>0.626*	*>0.153*		*>0.288*	*>0.884*	
**PP**						
Change d0-d7	-0.64 ± 3.21	-0.90 ±2.60	*0.370[3]*	-0.61 ± 3.49	-0.21 ± 2.37	*0.771[3]*
Change d0-d14	-0.82 ± 3.23	-1.06 ± 3.44	*0.751[3]*	-1.03 ± 2.64	0.58 ± 2.87	***0.038**[3]*
Change d0-d21	-0.30 ± 3.34	-0.68 ± 3.31	*0.483[3]*	-0.71 ± 2.65	0.24 ± 2.63	*0.151[3]*
Time effect[4]	*0.416*	*0.898*		*0.627*	*0.462*	
PSQI d0	10.03 ± 2.85	10.35 ±2.85	*0.636*	9.65 ± 3.40	9.45 ± 2.97	*0.812*
PSQI d7	9.39 ± 3.04	9.45 ± 3.24	*0.942*	9.33 ± 2.77	9.24 ± 3.54	*0.911*
PSQI d14	9.21 ± 2.76	9.29 ± 3.33	*0.919*	8.61 ± 2.58	10.03 ± 2.94	***0.045***
PSQI d21	9.73 ± 2.89	9.68 ± 3.03	*0.946*	8.94 ± 3.14	9.70 ± 3.15	*0.337*
Time effect[2]	*0.418*	*0.245*		*0.206*	*0.417*	
Post-hoc analysis	*>0.928*	*>0.374*		*>0.334*	*>0.411*	
**ModPP**						
Change d0-d7	-1.50 ± 3.20	-1.00 ± 2.77	*0.573*	-0.35 ± 2.95	-0.30 ± 2.42	*0.957*
Change d0-d14	-1.91 ± 3.06	-1,38 ± 3.79	*0.596*	-1.29 ± 1.90	0.83 ± 2.21	***0.001***
Change d0-d21	-1.57 ± 2.90	-1.13 ± 3.34	*0.633*	-0.79 ± 2.62	0.30 ± 2.87	*0.178*
Time effect	*0.764*	*0.835*		*0.203*	*0.116*	
Post-hoc analysis	*1.000*	*1.000*		*>0.347*	*>0.193*	
PSQI d0	11.35 ± 1.90	11.46 ± 2.06	*0.849*	10.38 ± 3.02	9.65 ± 2.96	*0.412*
PSQI d7	9.74 ± 3.29	10.46 ± 2.65	*0.413*	10.00 ± 2.68	9.35 ± 3.80	*0.504*
PSQI d14	9.43 ± 3.10	10.08 ± 3.28	*0.490*	9.08 ± 2.60	10.47 ± 2.37	*0.061*
PSQI d21	978 ± 3.10	10.33 ± 2.94	*0.536*	9.58 ± 3.09	9.96 ± 3.60	*0.704*
Time effect	***0.012***	*0.177*		*0.111*	*0.183*	
Post-hoc analysis	*0-14 days,* ***0.040***	*0-14 days 0.088*		*0-14 days* ***0.006***	*7-14 days 0.064*	

***Table S2**: Outcomes of the SmartSleep parameters per treatment group (A and B) and as measured at baseline and after 21 days of intervention.*

Treatment A started with the IP in the first intervention period, treatment B started with the placebo. DP: dairy-based test product. Placebo: skimmed milk powder.

		Treatment A: DP as the first product				Treatment B: Placebo as the first product				P values	
		DP		Placebo		DP		Placebo			
		Baseline	Day 21	Baseline	Day 21	Baseline	Day 21	Baseline	Day 21	Carry over effect	Efficacy
SOL	(min)	24.86 (18.99)	20.99(19.4)	27.89(29.13)	28.38(30.40)	30.84(24.99)	33.41(33.85)	31.79(20.93)	34.72(37.50)	0.661	0.483
WASO	(min)	39.58(19.70)	40.32(23.45)	38.73(24.99)	37.25(18.15)	42.36(33.62)	42.11(28.06)	39.32(20.48)	41.64(22.06)	0.731	0.569
TIB	(min)	480.25(48.39)	469.05(52.12)	479.83(48.75)	474.81(61.31)	464.92(58.99)	464.28(51.69)	465.13(53.38)	463.31(61.60)	0.343	0.557
TST	(min)	415.81(49.93)	407.74(47.98)	413.21(50.76)	409.18(62.00)	391.72(56.74)	388.77(56.63)	394.03(47.66)	386.95(63.03)	0.956	0.619
SE	(%)	86.5(5.6)	87.0(5.5)	86.2(7.3)	86.1(7.0)	84.4(7.7)	83.8(7.6)	84.8(5.0)	83.5(8.1)	0.289	0.998
REM	(min)	120.40(27.11)	117.15(28.33)	127.09(31.90)	117.13(31.22)	111.64(27.75)	106.25(25.76)	114.41(30.28)	107.25(20.81)	0.328	**0.049**
	(%)	25.0(4.8)	24.9(5.1)	26.6(6.7)	24.7(5.6)	24.1(4.9)	23.0(4.9)	24.6(5.0)	23.3(4.1)	0.810	0.163
NREM	(min)	295.41(42.30)	290.59(33.76)	288.13(43.10)	293.26(45.18)	280.08(46.40)	282.51(51.89)	279.61(42.95)	279.70(54.32)	0.857	0.417
	(%)	61.5(6.4)	62.1(5.2)	60.0(6.3)	61.7(5.5)	60.4(7.0)	60.8(8.1)	60.2(6.9)	60.2(7.5)	0.507	0.780
N2	(min)	220.44(36.25)	216.88(30.04)	214.04(34.80)	222.45(38.43)	210.87(43.02)	213.86(43.40)	215.47(39.73)	218.36(52.11)	0.934	0.403
	(%)	46.0(6.7)	46.5(6.2)	44.7(6.5)	47.1(7.0)	45.2(5.9)	45.9(6.3)	46.2(5.4)	46.8(7.1)	0.568	0.279
SWS (N3)	(min)	74.97(34.56)	73.71(28.12)	74.09(29.53)	70.81(32.28)	69.21(30.28)	68.66(29.89)	64.14(27.38)	61.34(29.30)	0.824	0.647
	(%)	15.5(6.5)	15.6(5.4)	15.3(5.4)	14.6(5.8)	15.1(6.7)	14.9(6.4)	14.0(6.0)	13.4(6.6)	0.754	0.702

Data are expressed as mean(SD) of the number of days per time point, or as % of total time in bed (TIB) or total sleep duration. Treatment group A, first consumed IP; treatment group B, first consumed placebo. Abbreviations: SOL: sleep onset latency (interval between lights off and first epoch sleep other than NREM phase 1); WASO: wake after sleep onset; TIB: time in bed; TST: total sleep time or total duration (sum of all sleep stages REM and NREM phases 1,2 and 3); SE: sleep efficiency (as % of TST); REM: rapid eye movement sleep; NREM: non-rapid eye movement (stages 1,2 and 3); N2: NREM sleep stage 2; SWS: slow wave sleep or NREM stage 3

Outcomes were evaluated using the method of Wellek et al (2012).

Wellek, S., & Blettner, M. (2012). Vom richtigen Umgang mit dem Crossover-Design in klinischen Studien: Teil 18 der Serie zur Bewertung wissenschaftlicher Publikationen. *Deutsches Arzteblatt International*, *109*(15), 276–281. https://doi.org/10.3238/arztebl.2012.0276

Table S3*: DASS 42 results (total and sub scores) per period and per group.*

Data are presented as mean ± SD and median (IQR). [1] Related Samples Friedman's 2-way analysis of variance by ranks. [2] Mann-Whitney U-test. DP: dairy-based test product; Placebo: skimmed milk powder; d: day; DASS-42: Depression Anxiety Stress Scale based 42 questions.

	**Period 1**		p-value period 1[2]	**Period 2**		p-value period 2[2]
	**DP**	**Placebo**		**DP**	**Placebo**	
**ITT**						
DASS total score d0	18.2 ± 1.3<br>16.0 (11)	16.9 ±11.9<br>14.0 (18.0)	*0.674*	17.6 ± 19.1<br>13.0 (15.5)	11.7 ± 8.7<br>10.0 (11.0)	*0.249*
DASS total score d21	12.9 ± 9.5<br>10.0 (9.0)	16.6 ± 13.5<br>13.0 (17.8)	*0.442*	17.0 ± 16.0<br>11.0 (22.3)	9.5 ± 8.1<br>8.0 (10.0)	*0.121*
Within group[1]; p	*0.090*	*0.567*		*0.587*	***0.011***	
DASS stress d0	7.4 ± 4.1<br>8.0 (6.0)	7.9 ± 5.2<br>8.5 (7.5)	*0.805*	7.4 ± 7.4<br>6.0 (7.5)	5.5 ± 3.6<br>6.0 (5.0)	*0.571*
DASS stress d21	6.3 ± 3.7<br>6.0 (6.0)	7.4 ± 5.5<br>6.5 (10.3)	*0.563*	7.2 ± 5.8<br>6.5 10.8)	4.4 ± 3.5<br>4.0 (5.0)	*0.078*
Within group[1]; p	*0.115*	*0.479*		*0.918*	***0.025***	
DASS anxiety d0	2.4 ± 2.8<br>1.0 (2.0)	2.9 ± 2.9<br>2.0 (2.0)	*0.421*	3.8 ± 5.2<br>2.0 (4.0)	2.0 ± 2.8<br>1.0 (3.0)	***0.022***
DASS anxiety d21	2.1 ± 2.5<br>1.0 (3.0)	2.8 ± 3.5<br>1.0 (3.3)	*0.449*	13.9 ± 4.0<br>2.0 (4.3)	1.5 ± 2.0<br>1.0 (2.0)	***0.008***
Within group[1]; p	*0.418*	*0.645*		*0.681*	*0.073*	
DASS depression d0	5.6 ± 6.8<br>3.0 (6.0)	6.1 ± 5.5<br>4.0 (8.5)	*0.447*	6.4 ± 7.8<br>3.5 (7.5)	4.1 ± 4.5<br>2.0 (6.0)	*0.228*
DASS depression d21	4.6 ± 5.6<br>3.0 (4.0)	6.4 ± 6.1<br>4.0 (10.5)	*0.170*	6.4 ± 7.6<br>4.0 (10.3)	3.7 ± 4.1<br>2.0 (4.0)	*0.332*
Within group[1]; p	*0.265*	*0.520*		*0.672*	*0.179*	
**PP**						
DASS total score d0	18.2 ± 11.3<br>16.0 (11.0)	17.0 ± 12.1<br>14.0 (18.0)	*0.830*	18.2 ± 19.9<br>14.0 (20.0)	11.8 ± 8.9<br>10.0 (10.5)	*0.279*
DASS total score d21	13.2 ± 9.6<br>10.0 (8.5)	16.6 ± 13.9<br>13.0 (20.0)	*0.577*	16.3 ± 15.9<br>11.0 (21.0)	9.8 ± 8.1<br>8.0 (10.0)	*0.236*
Within group[1]; p	*0.071*	*0.585*		*0.284*	***0.022***	
DASS stress d0	7.7 ± 4.0<br>8.0 (5.0)	8.0 ± 5.1<br>8.0 (7.0)	*0.936*	7.7 ± 7.6<br>6.0 (9.0)	5.7 ± 3.6<br>6.0 (5.0)	*0.599*
DASS stress d21	6.5 ± 3.7<br>6.0 (5.5)	7.4 ± 5.6<br>6.0 (10.0)	*0.721*	6.8 ± 5.6<br>6.0 (9.0)	4.6 ± 3.5<br>4.0 (5.0)	*0.186*
Within group[1]; p	*0.091*	*0.473*		*0.635*	***0.038***	
DASS anxiety d0	2.5 ± 2.8<br>2.0 (2.0)	2.8 ± 3.0<br>2.0 (2.0)	*0.705*	3.9 ± 5.5<br>2.0 (4.0)	2.2 ± 2.8<br>1.0 (3.5)	***0.058***
DASS anxiety d21	2.2 ± 2.6<br>1.0 (3.0)	2.9 ± 3.7<br>1.0 (4.0)	*0.680*	3.3 ± 3.9<br>2.0 (4.0)	1.5 ± 2.1<br>1.0 (2.0)	***0.035***
Within group[1]; p	*0.470*	*0.930*		*0.552*	*0.073*	
DASS depression d0	5.6 ± 7.0<br>3.0 (7.0)	6.3 ± 5.7<br>4.0 (9.0)	*0.369*	6.6 ± 8.1<br>4.0 (9.0)	3.9 ± 4.5<br>2.0 (5.5)	*0.209*
DASS depression d21	4.5 ± 5.6<br>3.0 (4.0)	6.4 ± 6.2<br>4.0 (11.0)	*0.237*	6.2 ± 7.7<br>4.0 (10.0)	3.6 ± 4.1<br>2.0 (4.0)	*0.426*
Within group[1]; p	*0.195*	*0.563*		*0.329*	*0.340*	
**modPP**						
DASS total score d0	8.8 ± 3.6<br>9.0 (5.0)	18.2 ± 12.2<br>14.5 (16.0)	*0.815*	20.0 ± 21.7<br>14.5 (20.0)	13.3 ± 9.8<br>12.0 (11.0)	*0.382*
DASS total score d21	15.3 ± 10.5<br>11.0 (18.0)	17.9 ± 13.9<br>13.5 (23.3)	*0.647*	17.4 ± 16.7<br>11.0 (21.5)	10.8 ± 8.8<br>10.0 (10.0)	*0.400*
Within group[1]; p	*0.097*	*0.738*		*0.217*	***0.033***	
DASS stress d0	8.8 ± 3.6<br>9.0 (5.0)	8.5 ± 4.6<br>9.0 (6.8)	*0.748*	8.3 ± 8.2<br>6.0 (8.0)	6.4 ± 3.5<br>6.0 (6.0)	*0.847*
DASS stress d21	7.6 ± 3.4<br>8.0 (5.0)	8.0 ± 5.6<br>7.5 (11.0)	*0.957*	7.0 ± 5.5<br>6.5 (8.8)	4.8 ± 3.6<br>4.0 (5.0)	*0.211*
Within group[1]; p	*0.166*	*0.587*		*0.418*	***0.026***	
DASS anxiety d0	2.5 ± 2.6<br>2.0 (2.0)	3.1 ± 3.1<br>2.0 (2.0)	*0.593*	4.4 ± 6.0<br>2.0 (4.8)	2.2 ± 3.2<br>1.0 (4.0)	***0.045***
DASS anxiety d21	2.4 ± 2.9<br>1.0 (4.0)	2.9 ± 3.7<br>1.0 (5.0)	*0.810*	3.6 ± 4.3<br>2.0 (4.8)	1.4 ± 1.9<br>1.0 (2.0)	***0.041***
Within group[1]; p	*0.676*	*0.519*		*0.667*	*0.109*	

DASS depression d0	6.9 ± 7.7<br>4.0 (8.0)	6.5 ± 5.8<br>4.5 (8.8)	*0.923*	7.4 ± 8.7<br>5.5 (9.5)	4.7 ± 5.0<br>2.0 (6.0)	*0.319*
DASS depression d21	5.3 ± 6.4<br>3.0 (7.0)	7.0 ± 6.3<br>5.5 (10.8)	*0.280*	6.8 ± 8.3<br>4.0 (10.0)	4.6 ± 4.6<br>3.0 (7.0)	*0.722*
Within group[1]; p	*0.093*	*0.251*		*0.359*	*0.640*	

Table S4*: Early morning saliva cortisol values per treatment. Saliva was sampled starting at waking-up and after that every 15 minutes up till 1 hours after waking up.*

Data are presented as mean ± SD; median (IQR). [1] Mann-Whitney U-test. [2] Independent samples T-test. DP: dairy-based test product; Placebo: skimmed milk powder; d: day.

	**Intervention periods 1+2**		p-value
	**DP**	**Placebo**	
**ITT**			
Cortisol d0, 0 min	3.36±1.54; 3.20 (2.20)	3.38±1.44; 3.05 (2.18)	*0.973*
Cortisol d0, 15 min	4.16±1.90; 4.00 (2.00)	4.27±1.81; 4.00 (2.30)	*0.997*
Cortisol d0, 30 min	4.19±2.15; 3.60 (2.70)	4.54±2.13; 4.05 (3.00)	*0.343*
Cortisol d0, 45 min	3.79±1.95; 3.20 (2.70)	3.94±1.73; 3.65 (2.48)	*0.500*
Cortisol d0, 60 min	3.33±1.89; 2.90 (1.70)	3.44±1.57; 3.15 (2.35)	*0.318*
Cortisol d21, 0 min	3.19±1.45; 3.00 2.20)	3.78±1.78; 3.50 (1.85)	***0.045*** ***0.031*** [2]
Cortisol d21, 15 min	3.76±1.70; 3.80 (2.10)	4.24±1.73; 4.25 (1.95)	*0.074*
Cortisol d21, 30 min	3.91±1.89; 3.70 (2.80)	4.26±1.89; 4.20 (2.48)	*0.228*
Cortisol d21, 45 min	3.58±1.84; 3.40 (2.40)	3.82±1.88; 3.40 (2.65)	*0.527*
Cortisol d21, 60 min	3.23±1.70; 2.80 (2.40)	3.40±1.72; 2.95 (2.13)	*0.481*
**PP**			
Cortisol d0, 0 min	3.38±1.56; 3.20 (2.10)	3.34±1.44; 3.00 (2.05)	*0.784*
Cortisol d0, 15 min	4.20±1.87; 4.10 (2.00)	4.24±1.83; 3.90 (2.30)	*0.767*
Cortisol d0, 30 min	4.14±2.06; 3.60 (2.70)	4.52±2.08; 4.10 (2.85)	*0.317*
Cortisol d0, 45 min	3.71±1.79; 3.20 (2.50)	3.89±1.57; 3.60 (2.35)	*0.463*
Cortisol d0, 60 min	3.17±1.60; 2.90 (1.70)	3.40±1.49; 3.10 (2.25)	*0.245*
Cortisol d21, 0 min	3.21±1.45; 3.20 (2.20)	3.83±1.82; 3.50 (1.95)	*0.059* ***0.031*** [2]
Cortisol d21, 15 min	3.90±1.66; 3.90 (1.90)	4.30±1.76; 4.40 (2.00)	*0.161*
Cortisol d21, 30 min	4.03±1.87; 3.80 (2.60)	4.32±1.91; 4.20 (2.40)	*0.347*
Cortisol d21, 45 min	3.67±1.84; 3.50 (2.30)	3.86±1.93; 3.40 (2.85)	*0.726*
Cortisol d21, 60 min	3.31±1.68; 2.90 (2.30)	3.40±1.76; 2.90 (2.15)	*0.702*
**modPP**			
Cortisol d0, 0 min	3.38±1.64; 3.10 (1.90)	3.38±1.46; 3.10 (2.15)	*0.788*
Cortisol d0, 15 min	4.27±1.96; 4.10 (2.30)	4.15±1.67; 4.00 (2.30)	*0.650*
Cortisol d0, 30 min	4.23±2.11; 3.60 (2.70)	4.55±1.90; 4.30 (2.80)	*0.370*
Cortisol d0, 45 min	3.78±1.75; 3.40 (2.10)	3.94±1.55; 3.80 (2.35)	*0.586*
Cortisol d0, 60 min	3.15±1.37; 3.00 (1.60)	3.44±1.46; 3.10 (1.85)	*0.272*
Cortisol d21, 0 min	3.03±1.36; 2.90 (2.10)	3.90±1.91; 3.60 (2.05)	***0.033*** ***0.031*** [2]
Cortisol d21, 15 min	3.97±1.81; 4.00 (1.90)	4.42±1.78; 4.50 (2.00)	*0.193*
Cortisol d21, 30 min	4.21±2.02; 3.80 (3.10)	4.50±2.03; 4.20 (2.50)	*0.499*
Cortisol d21, 45 min	3.86±1.89; 3.70 (3.30)	4.09±2.11; 3.60 (3.00)	*0.846*
Cortisol d21, 60 min	3.51±1.70; 3.20 (2.40)	3.55±1.85; 3.10 (2.65)	*0.879*

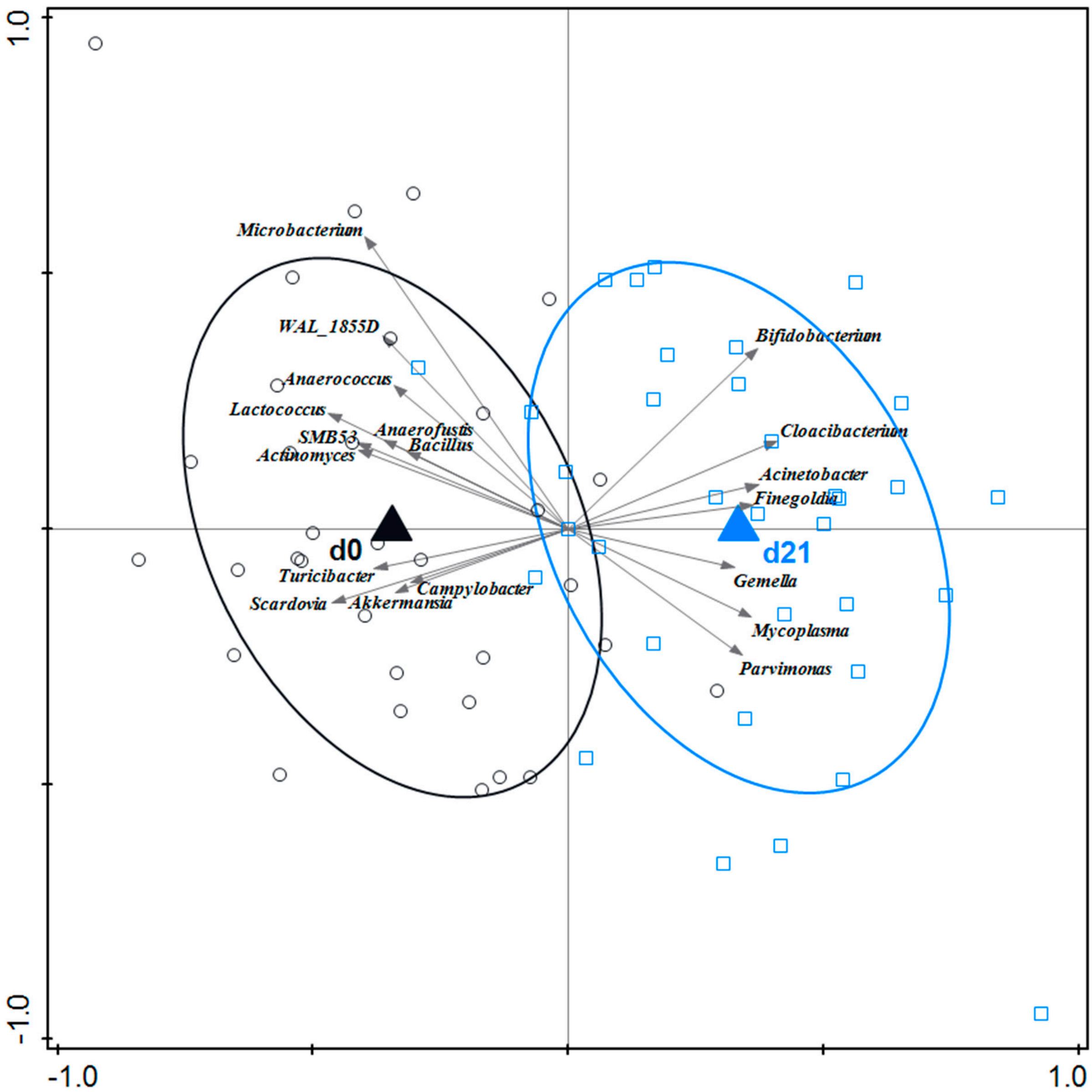

Supplemental Figure S1. RDA on the genus level, assessing the effect of time on gut microbiota composition within the DP group. The covariance attributable to subject and PCR protocol was first fitted by regression and then partialled out (removed) from the ordination. Genera were used as response data and time point was explanatory data. Variation explained by time point was 3.0%, p=0.004.

RDA: redundancy analysis. DP: dairy-based product.

Supplemental White paper

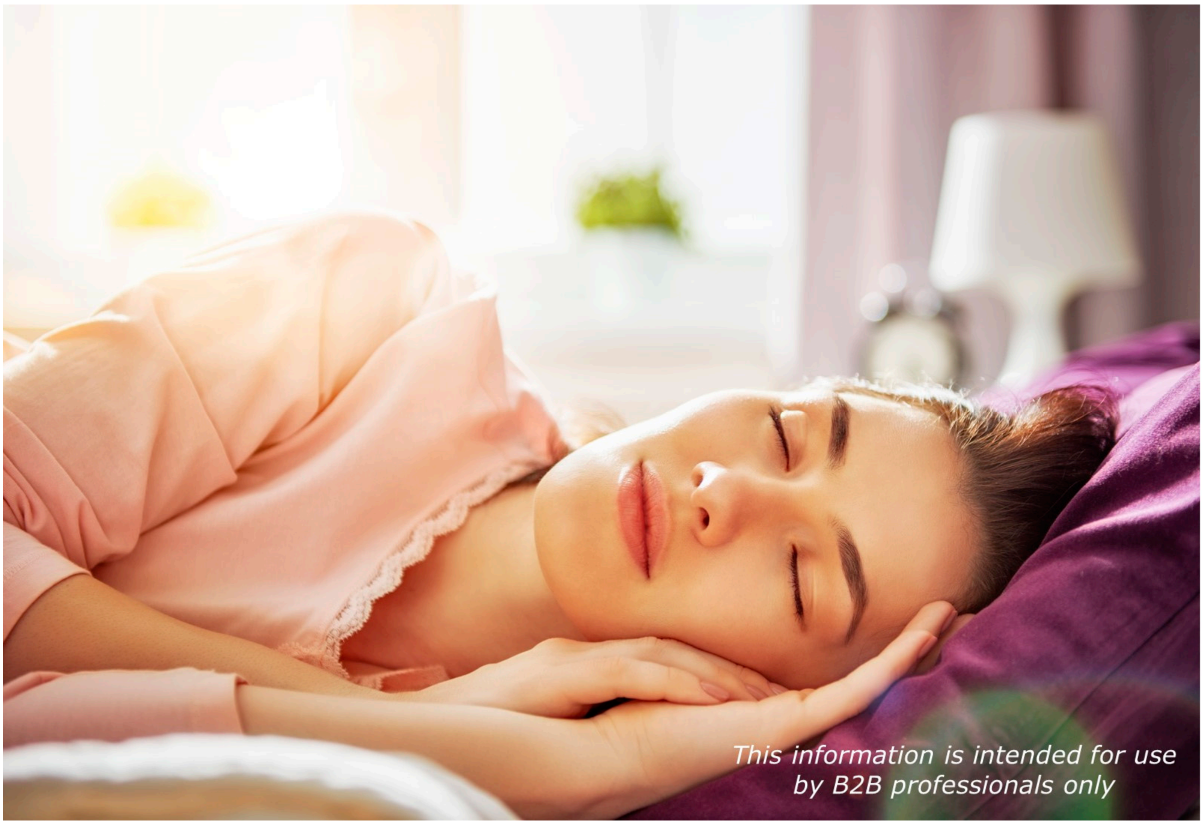

White paper

Support qualitative sleep in adults: a positive indication from a consumer test

Outcomes consumer test: **'21-day sleep challenge'**

Authors: Anne Schaafsma, Iris Hofman, Astrid Horstman, Leonard Mallee
FrieslandCampina Innovation Center

Date: September 2018

Introduction

Sleep is really important for optimal human health. However sleep disorders have become a major public health concern [1]. There are several underlying mechanisms for sleep disorders, such as anatomical or genetic factors, which could influence the sleep quality. Besides these underlying mechanisms and also environmental factors, stress has a big impact on sleep quality [2]. A consumer test (*21 day challenge)* was performed to get a feeling of the possible effect of a formula (Table 1) on sleep quality in apparently healthy people, who were recruited via an advertisement addressing the improvement of sleep.

Insomnia (sleeplessness) has been defined by[3][4]

- Difficulty initiating sleep
- Difficulty maintaining sleep
- Non-restorative sleep
- Accompanied by decreased daytime functioning

Examples of decreased daytime functioning due to insomnia [3][4]

- Feeling fatigue/malaise
- Daytime sleepiness
- Mood disturbance/irritability
- Motivation/energy/initiative reduction
- Attention/concentration/memory impairment

Primary and secondary outcome measurements

The primary outcome was the change in overall sleep quality after 21 days of daily consumption of the test blend as measured by the Pittsburgh Sleep Quality Index (PSQI). The items include hours of sleep, ratings for frequency of sleep concerns, general sleep quality and daytime factors related to poor sleep [5]. The Secondary outcomes were wake up fresh and mood measured by a Visual Analogue Scale (VAS) and experience of feeling fit measured by a rating scale from 0 – 10.

Materials and methods

Study design

The consumer test was initiated by and monitored at the FrieslandCampina Innovation Centre in Wageningen between June 2018 and September 2018. An overview of inclusion and study activities is shown in **Figure 1**.

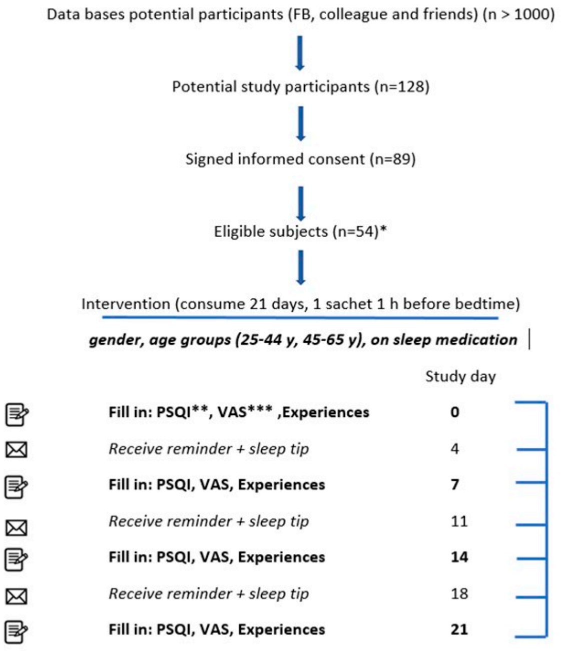

Figure 1 Study design. ** 54 subjects were eligible for statistical analysis. These participants completed all questionnaires at day 0, 7, 14 and 21.**PSQI = Pittsburg Sleep Quality Index, ***VAS = Visual Analogue Scale.*

Study population

Potential participants were recruited via social media or among colleagues with an advertisement targeted at studying the improvement of sleep. The only exclusion criteria was lactose intolerance.

Study treatment

The test product was a powder blend (**Table 1**), and was personally provided as 21 sachets + one spare sachet in 2 boxes. No development was done on taste and smell. The participants were required to take one sachet, mix the powder with 150 ml water, stir it, and consume the dissolved product directly, preferably 1h before going to bed.

Table 1. Ingredients formula

Test sachet 21-day challenge	Total amount:
**Ingredients**	16,3 g
Milk protein	
Fibers	
Milk protein peptides	
Vitamins	
Minerals	

The 21 days challenge took place at home, and participants were not restricted in any kind of medication or to behavior. Assessments of sleep quality, mood, stress and feeling fit were completed by the participants via an online questionnaire at baseline (day 0), and after 7, 14 and 21 days. In this test no reference or placebo effect was used.

Statistical analysis

All measures were analyzed using IBM SPSS Statistics (version 24, IBM Corp., Armonk, USA). A generalized estimating equations (GEE) was used to analyze the change of the PSQI, VAS A, VAS B, and the fit score in the course of time. The level of significance was set at 5%. Gender, age and sleep medication (component 6 of the PSQI [14]) were included as confounding variables. Time in days was included in the analysis as a continuous or categorical variable.

Results

Primary outcome

Over the 21 days test period, the participants (n=54) showed a significant reduction (from 11.20 ± 0.56 to 7.85 ± 0.55, p<.000)* in mean total PSQI score from baseline to end of treatment (day 21) (see figure 1). A lower PSQI means an improvement of sleep quality. No significant differences were found between gender and age. No significant reduction of the PSQI score was found in participants on sleep medication. The mean change in total PSQI across time is presented in **figure 2**. The response rate for the VAS was too low to draw conclusion, therefore this data will not be described in the report.

Table 2. Participant characteristics included in evaluation (n=54)

Characteristics	
**Male (M), n (%)**	24 (44%)
**Female (F), n (%)**	30 (56%)
**Age group**	
25-44	N = 23 (F=14, M=9)
45-65	N = 31 (F=16, M=15)
**Sleep medication during challenge n, (%)**	8 (15%)
**Medication during challenge**	
25 - 44	N = 3 (F=2, M=1)
45 – 65	N = 5 (F=3, M=2)

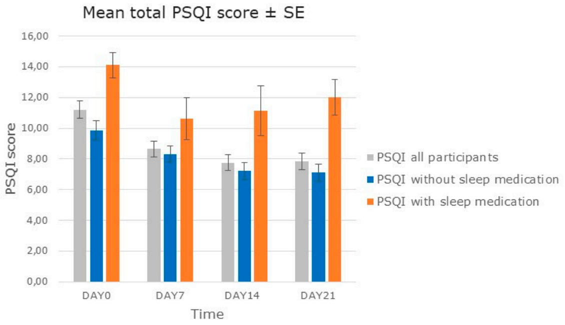

Figure 2. The mean total PSQI scores and SE over the 21-day. *Divided into all participants included (gray bars), participants without sleep medication (blue bars) and participants using sleep medication (orange bars) during 21-day challenge. *significant decrease of PSQI score over 21 days (p<.05) as compared to baseline*

Discussion

This study provides an indication of the possible positive effect of a formula. 'Possible' since there may be a placebo effect (which was not measured). This indication is based on the PSQI improvement over a 21 days treatment period in participants without using sleep medication. Although the total mean PSQI score after treatment still gives an indication for poor sleep quality (PSQI score >5 indicates a reduced sleep quality[5]), a decrease of $\Delta 3.1$ is similar to what have been found in previous studies. Nielson et al. (2010) found a decrease in PSQI from 10.4 ± 0.4 to 7.0 ± 0.4 [6] after supplementation of 320 mg magnesium to 100 (22 males, 78 females) participants with poor sleep quality (PSQI > 5). Besides Saint-Hilaire et al. (2009) found an improvement of Δ3.5 of the PSQI score in a study with 32 (7 males, 25 females) participants with a PSQI > 4, of which 20 consumed the product and 12 a placebo. The significant improvement was found after 28 days of treatment with 2.70 mg Lactium added to the treatment product[7]. No gender or age differences in the effect of the current formula were found. The response rates for the VAS scores were too low to draw conclusions. Unfortunately the formula had no effect on participants using sleep medication during the treatment period. The current consumer test had some limitations that could have affected the results. Firstly the questionnaires were on a voluntary basis resulting in a number of non-responders and therefore some missing data. Secondly no personal contact occurred during the assessments, and as a result information from drop-outs is missing. And finally the absence of a placebo group.

Conclusion

Taking all limitations into account this consumer test indicates a possible beneficial effect of the formula on sleep quality as measured by PSQI score in apparently healthy subjects but without medication.

Based on the mentioned limitations a suggestion for a follow up research would be to use online questionnaires only for increasing the response rate and to incorporate a placebo group in the study. Furthermore it could be of interest to substantiate the subjective questionnaires with a sleep tracker to measure more objective sleep parameters.

References

1. Ford, E. S., Wheaton, A. G., Cunningham, T. J., Giles, W. H., Chapman, D. P., & Croft, J. B. (2014). Trends in outpatient visits for insomnia, sleep apnea, and prescriptions for sleep medications among US adults: Findings from the national ambulatory medical care survey 1999-2010. *Sleep*, 37(8), 1283–1293
2. Han, K. S., Kim, L., & Shim, I. (2012). Stress and sleep disorder. Experimental neurobiology, 21(4), 141-150
3. Sateia, M. J. (2014). International classification of sleep disorders. Chest, 146(5), 1387-1394.;.
4. Baglioni, C., Spiegelhalder, K., Lombardo, C., & Riemann, D. (2010). Sleep and emotions: a focus on insomnia. *Sleep medicine reviews*, *14*(4), 227-238..
5. Buysse, D. J., Reynolds III, C. F., Monk, T. H., Berman, S. R., & Kupfer, D. J. (1989). The Pittsburgh Sleep Quality Index: a new instrument for psychiatric practice and research. *Psychiatry research*, *28*(2), 193-213.
6. Nielsen, F. H., Johnson, L. K., & Zeng, H. (2010). Magnesium supplementation improves indicators of low magnesium status and inflammatory stress in adults older than 51 years with poor quality sleep. Magnesium Research, 23(4), 158-168.
7. de Saint-Hilaire, Z., Messaoudi, M., Desor, D., & Kobayashi, T. (2009). Effects of a bovine alpha S1-Casein tryptic hydrolysate (CTH) on sleep disorder in Japanese general population. Open Sleep Journal, 2, 26-32
